# Velopharyngeal Inadequacy-Related Quality of Life Assessment: The Instrument Development and Application Review

**DOI:** 10.3389/fsurg.2022.796941

**Published:** 2022-03-08

**Authors:** Nan Chen, Bing Shi, Hanyao Huang

**Affiliations:** ^1^Department of Epidemiology and Health Statistics, West China School of Public Health and West China Fourth Hospital, Sichuan University, Chengdu, China; ^2^State Key Laboratory of Oral Diseases & National Clinical Research Center for Oral Diseases and Department of Oral Maxillofacial Surgery, West China Hospital of Stomatology, Sichuan University, Chengdu, China

**Keywords:** velopharyngeal inadequacy, quality of life, instruments, patient-report outcomes, patient-report outcome questionnaire

## Abstract

**Objective:**

For the patient-reported outcome (PRO) measures related to patients with velopharyngeal inadequacy (VPI), different quality of life (QOL) instruments have been developed. The present systematic review was designated to identify current VPI-related QOL instrument development, validation, and applicability.

**Methods:**

Pubmed, Cochrane, Embase, Web of Science, and EBSCO databases were searched in January 2022. “Velopharyngeal” or “palatopharyngeal” and “quality of life” or “life quality” were searched in title, abstract, and keywords. This study followed Preferred Reporting Items for Systematic Review and Meta-analysis (PRISMA) guidelines. Two investigators independently reviewed abstracts and full texts of the identified literature. An established checklist was used to evaluate the measurement properties of each identified instrument.

**Results:**

A total of 375 articles and 13 instruments were identified, which can be divided into nine types of families according to their development procedures. Developmental and measurement characteristics, evidence of conceptual model, content validity, reliability, construct validity, scoring, interpretation, respondent burden, and presentation for all instruments were shown.

**Conclusion:**

The patient's self-report assessment and parent-proxy assessment are both valuable. The conclusion that any QOL instrument is absolutely the best for patients with velopharyngeal inadequacy could not be drawn. Understanding the development and characteristics of different QOL instruments, including their reliability, validity, aim, target, language, and resource, should be important before application in clinic or research.

## Introduction

Velopharyngeal inadequacy (VPI) is the generic term for denoting three types of abnormal velopharyngeal function, namely, velopharyngeal insufficiency which is caused by structural etiologies, velopharyngeal incompetency which is incurred with neurogenic etiologies, and velopharyngeal mislearning which is related to functional etiologies (1). Velopharyngeal inadequacy occurs at high frequency among patients with post-operative cleft palate and patients with non-cleft palate functional velopharyngeal inadequacy, both of which are caused by multiple reasons. The causes can be divided into congenital and acquired (Table 1) (2–9). As orofacial clefts, like cleft palate, are among one of the most common congenital disabilities worldwide (10, 11), the problems caused by velopharyngeal inadequacy, such as speech and swallowing problems, remain a significant challenge to clinicians.

**Table 1:** Causes of velopharyngeal inadequacy.

**Congenital causes**	**Acquired cuases**
1. Submucous cleft palate	1. Palatoplasty
2. Van Der Woude syndrome	2. Le Fort I maxillary advancement
3. Kallmann syndrome	3. Adenoidectomy
4. Pierre Robin sequence	4. Adenotonsillectomy
5. DiGeorge syndrome	5. Tonsillectomy
6. Kabuki makeup	6. Uvulopalatopharyngoplasty
7. Hemifacial microsomia	7. Trauma
8. Klippel–Feil syndrome	8. Oral or pharyngeal cavity tumors
9. Down syndrome	
10. Mosaic trisomy 8	
12. Irregular adenoids	
12. Hypertrophic tonsils	
13. Velocardiofacial syndrome	
14. Unilateral hypoplasia of the velum and pharynx	

Speech therapy, prosthetic appliances, and surgery can help restore the velopharyngeal inadequacy (12). Objective measurements like nasopharyngoscope and imaging evaluation for the measurement of velopharyngeal gap size and nasalance are usually used for post-operative assessment. However, anatomic change and improvement cannot guarantee functional recovery, let alone solve social and emotional problems that come with the disability. Speech evaluation is commonly applied for function tests which are mostly based on the experience of speech therapists. It is possible to use the automatic evaluation system to assist in diagnosing specific speech problems (13, 14). Despite this, speech evaluation still cannot illustrate the feelings of the patients.

Tools are strongly needed in understanding the patients' perceptions. For the patient-reported outcome (PRO) measures related to patients with velopharyngeal inadequacy, different quality of life (QOL) instruments have been developed during the past two decades. However, clinicians or researchers may find it challenging to choose the appropriate instrument for their study and presume that published instruments all have appropriate measurement properties. A checklist developed by Francis DO was designed to help identify components that are considerably crucial to the construction of PRO measures. This particular checklist was applied to evaluate VPI-related QOL instruments in this study (Table 2) (15). This study aims to perform a comprehensive review of VPI-related QOL instruments and provide a pragmatic approach to assessing the QOL of patients with velopharyngeal inadequacy.

**Table 2:** Checklist of key characteristics to consider when evaluating a patient-reported outcome (PRO) measure^a^.

**Characteristic**	**Score**
**Conceptual model**	
1. Has the PRO construct to be measured been specifically defined?	
2. Has the intended respondent population been described?	
3. Does the conceptual model address whether a single construct or scale or multiple subscales are expected?	
**Content validity**	
4. Is there evidence that members of the intended respondent population were involved in the PRO measure's development?	
5. Is there evidence that content experts were involved in the PRO measure's development?	
6. Is there a description of the method by which items or questions were determined?	
**Reliability**	
7. Is there evidence that the PRO measure's reliability was tested?	
8. Are reported indexes of reliability adequate?	
**Construct validity**	
9. Is there reported quantitative justification that a single scale or multiple subscales exist in the PRO measure?	
10. Is the PRO measure intended to measure change over time? If yes, is there evidence of both test-retest reliability and responsiveness to change? Otherwise, award 1 point if there is an explicit statement that the PRO measure is not intended to measure change over time.	
11. Are there findings supporting expected associations with existing PRO measures or with other relevant data?	
12. Are there findings supporting expected differences in scores between relevant known groups?	
**Scoring and interpretation**	
13. Is there documentation of how to score the PRO measure?	
14. Has a plan for managing or interpreting missing responses been described?	
15. Is information provided about how to interpret the PRO measure scores?	
**Respondent burden and presentation**	
16. Is the time to complete reported and reasonable? Or, if it is not reported, is the number of questions appropriate for the intended application?	
17. Is there a description of the literacy level of the PRO measure?	
18. Is the entire PRO measure available for public viewing?	

*^a^Instructions: Please indicate in the Score column whether or not the information provided in the citation or source document meets each criterion (0 indicates criterion not met and 1 indicates criterion met)*.

## Materials and Methods

This review was conducted with reference to the Preferred Reporting Items for Systematic Review and Meta-analysis (PRISMA) guidelines (16). The research protocol was censored and approved by the Ethic Committee of West China Hospital of Stomatology, Sichuan University (Approval No. WCHSIRB-D-2016-084R1).

### Search Strategy

A comprehensive search was conducted through literature databases, including Pubmed, Cochrane, Embase, Web of Science, and EBSCO host. The search of literature was conducted in January 2022. No publication date limit was set during the literature search. “velopharyngeal” or “palatopharyngeal” and “quality of life” or “life quality” were searched in title, abstract, and keywords.

### Study Selection

Abstracts for all studies identified in the literature search were independently reviewed by two investigators. Those meeting the predetermined screening criteria were advanced to full-text review. Inclusion criteria were as follows: 1. Research is on human subjects; 2. The participants include the patients with velopharyngeal inadequacy; and 3. The study mentioned at least one kind of instrument for QOL. Articles lacking adequate information in their title or abstract for determining eligibility were also included in the full-text review phase. Only the articles describing the development and validation of each instrument with the original version were included for analysis, and the translated versions or modified versions were excluded.

### Data Extraction and PRO Measures Assessment

First, one reviewer assessed each study's methods using a criteria checklist developed *a priori* (15). Another reviewer completed the evidence table which has been thoroughly discussed between the three authors to compare the characteristics of QOL measurements. Then, the two reviewers checked each other's results and dealt with the ambiguities. If they were unable to reach a consensus, the third author was consulted.

The checklist was designed to help reviewers identify components crucial to constructing patient-reported outcome (PRO) measures. Measurement properties, including conceptual model, content validity, reliability, construct validity, scoring and interpretation, respondent burden, and presentation for all instruments were evaluated.

### Data Synthesis

Meta-analysis was not applicable for data aggregation due to the heterogeneity of studies in constructs, methods, and intended purposes. Efforts were still made to summarize some useful regular patterns for clinical practice.

## Results

### Literature Search and Screening

**Figure 1:**
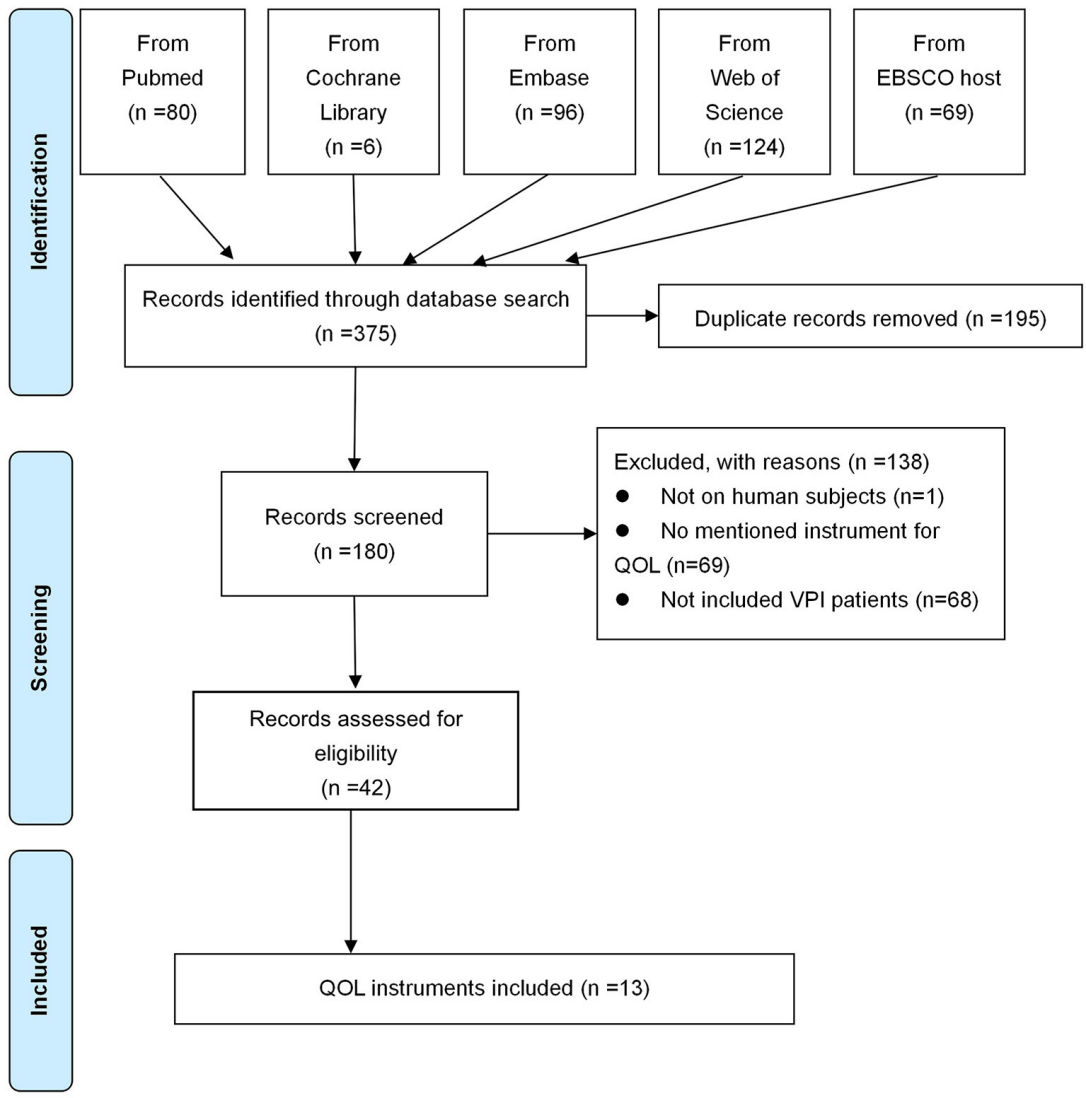
Literature searching and screening flow diagram. QOL, quality of life; VPI, velopharyngeal insufficiency.

**Figure 2:**
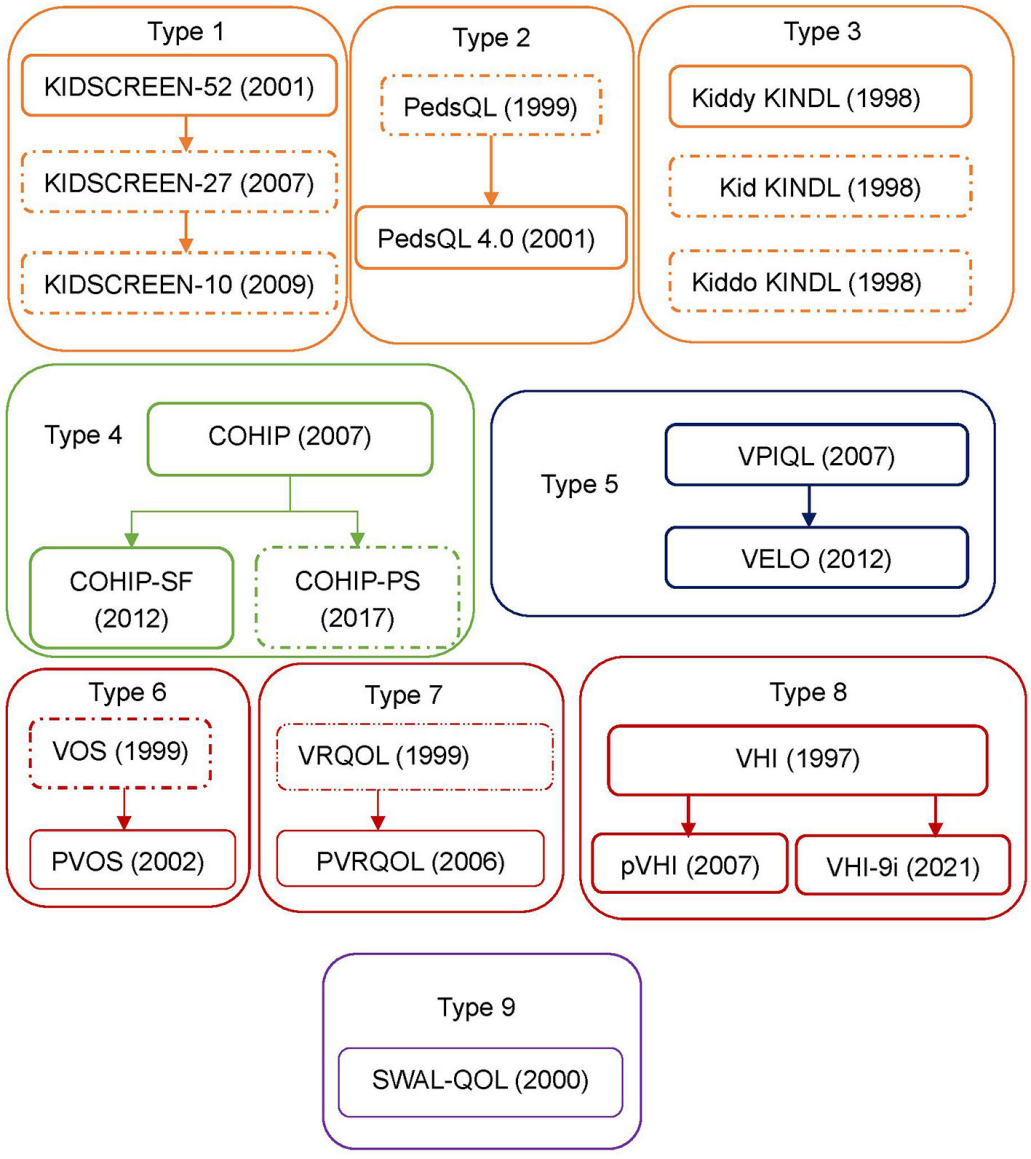
Instruments types applied for VPI patients. Development procedures and types of the instrument applied to patients with velopharyngeal inadequacy. Thirteen instruments that have been applied to patients with velopharyngeal inadequacy are identified. These can be divided into 9 types according to the development procedure. A dotted box would mean that the instrument has not been applied to patients with velopharyngeal inadequacy. PedsQL, pediatric quality of life inventory; PedsQL 4.0, pediatric quality of life inventory 4.0 generic core scales; KINDL, german generic quality of life instrument for children; COHIP, child oral health ompact profile; COHIP-SF, child oral health ompact profile-short form; COHIP-PS, child oral health ompact profile-preschool version; VPIQOL, velopharyngeal insufficiency quality of life; VELO, velopharyngeal insufficiency effrets of the outcomes instrument; VOS, voice outcome survey; PVOS, pediatric voice outcome survey; VRQOL, voice-related quality-of-life survey; PVRQOL, pediatric voice-related quality-of-life survey; VHI, voice handicap index; pVHI, pediatric voice handicap index; VHI-9i, 9-item voice handicap index; SWAL-QOL, swallowing quality of life questionnaire.

The literature search and screening flow diagram were shown in Figure 1 (16). A total of 375 articles were identified. After exclusion of duplicates, 180 articles remained. Eventually, 13 instruments were identified, including KIDSCREEN, PedsQL 4.0 Generic Core Scales (Pediatric Quality of Life Inventory 4.0), KINDL, Child Oral Health Impact Profile (COHIP), Child Oral Health Impact Profile-Short Form (COHIP-SF), Velopharyngeal Insufficiency Quality of Life (VPIQOL), Velopharyngeal Insufficiency Effects on Life Outcomes instrument (VELO), Pediatric Voice Outcome Survey (PVOS), Pediatric Voice-Related Quality-of-Life survey (PVRQOL), Voice Handicap Index (VHI), Pediatric Voice Handicap Index (pVHI), 9-item Voice Handicap Index (VHI-9i), and Swallowing Quality of Life questionnaire (SWAL-QOL). According to the development procedure, these instruments could be divided into nine types of families (Figure 2). Among these instruments, only VPIQOL and VELO were specifically designed for patients with velopharyngeal inadequacy (17–20).

### Developmental and Measurement Characteristics

The number of participants involved in different studies ranged from 29 to 22,295 (Supplementary Table 1). In the instrument development, the proportion of females was the lowest for SWAL-QOL (21.5%), followed by PVRQOL with 40%. The percentage of females in VHI development was the highest (60.32%). The other instruments had balanced proportions of females and males at around 50%. A total of 10 instruments were designed for children and their parents, in which the mean age of patients ranged from 8.7 to 13.2. Three instruments were designed without age restriction, in which the mean age of participants ranging from 45 to 66.1. The United States of America (USA) had an active role in developing instruments, and participated in the development of 11 instruments. Multiple European countries jointly developed KIDSCREEN and VHI-9i, while the KINDL instrument originated from Germany.

These data regarding the developmental characteristics of instruments could be used for the quality evaluation of evidence (Supplementary Table 1). Type 1 to type 3 instruments, including KIDSCREEN, PedsQL 4.0, and KINDL, were designed for the general health of pediatrics and they can be used on healthy children and children with acute or chronic diseases (21–23). Type 4 instruments (COHIP and COHIP-SF) aimed to assess the oral-facial wellbeing of school-age children (24–29). Type 5 instruments (VPIQL and VELO) were developed for patients with velopharyngeal inadequacy (17, 18). Type 6 to type 8 instruments (PVOS, PVRQOL, VHI families) focused on dysphonia problems (30–36). Lastly, type 9 instruments (SWAL-QOL) concentrated on dysphagia problems (37–39). Due to the different study populations, the distribution of pathology and mean age were varied between different types. The sample of KIDSCREEN, which came from a European project called “Screening and Promotion for Health-related Quality of Life in Children and Adolescents - A European Public Health Perspective” that included 13 European countries, was the biggest among all the instruments. The sample of PedsQL 4.0, which came from the State's Children's Health Insurance Program (SCHIP), was the second largest. Other instrument data were hospital-based, and only KIDSCREEN, PedsQL 4.0, and COHIP-SF were population-based.

Measurement aims, target populations, and item characteristics of these instruments were shown in Table 3. As a measure of PRO, QOL was evaluated based on the patients' experience and perception. For many patients with velopharyngeal inadequacy, the causes are congenital (Table 1) (11). Considering the development of their cognition, it was hard for young children to evaluate their QOL by themselves. Hence, the caregivers' proxy QOL assessment was essential for such situations. Here, the concept “patient” in PRO did not only refer to the patients' selves, but also included the parents. In the usage of the QOL instruments for patients with velopharyngeal inadequacy, the youngest children ranged from 2 (PedsQL 4.0, PVOS and PVRQOL) (22, 30, 31) to 3 years old (VELO and pVHI) (18, 36). However, for those self-report QOL instruments for VPI, the youngest age was 4 years old (KINDL) (23), followed by 5 (PedsQL 4.0 and VPIQOL) (17, 22) and 8 years old (KIDSCREEN, VELO, COHIP, COHIP-SF) (18, 21, 24, 29).

**Table 3:** Measurement aims, target populations, and item characteristics of instruments for the quality of life (QOL) of patients with velopharyngeal inadequacy.

**Instrument**	**Development year**	**Measurement aim**	**Target population**	**Language version**	**No. of Items or domains**	**Response options**	**Domains**	**Reporter and versions**
KIDSCREEN	2001	To assess children's and adolescents' subjective health and wellbeing	Healthy and chronically ill children and adolescents from 8 to 18 years	English German Dutch Spanish Portuguese French Czech Polish Hungarian Swedish Greek Persian Japanese Italian Korean Latvian Russian	KIDSCREEN-52: 52 items and 10 domains KIDSCREEN-27: 27 items and 5 domains KIDSCREEN-10: 10 items and 1 domain	5-point Likert-type	Physical-, psychological wellbeing, moods and emotions, self-perception, autonomy, parent relations and home life, social support and peers, school environment, social acceptance (bullying), financial resources	Children self-report: KIDSCREEN-52 KIDSCREEN-27 KIDSCREEN-10 Parent proxy-report: KIDSCREEN-52 KIDSCREEN-27 KIDSCREEN-10
PedsQL 4.0 (PedsQL 4.0 generic core scales)	2001	To measure health related quality of life in children and adolescents ages 2–18	Healthy school and community populations, as well as pediatric populations with acute and chronic health conditions between 2 and 18	English UK-English Spanish Japanese Chinese Thai Hungarian Brazilian Korean and so on (Total of 92 kinds of languages)	Core: 23 items, 4 domains	5-point Likert-type	Physical functioning, Psychosocial health: emotional functioning, social functioning, school functioning	Child self-report: 5–7 years; 8–12 years; 13–18 years. Parent proxy-report: 2–4 years; 5–7 years; 8–12 years; 13–18 years.
KINDL (Generic core instrument)	1998	To access health-related quality of life in children and adolescents aged 3 years and older	Healthy and ill children and adolescents between 3 and 17 years of age	Abrabic Bulgarian Chinese Danish Dutch English French German Greek Iranian Italian Japanese Korean Nepalese Norwegian Polish Portuguese Russian Serbian Sinhala Spanish Swedish Turkish Vietnamese	The original one had 40 items and 4 domains. The latest one has 19–24 items and 6 domains for children, 24–46 items and 6 domains for parents.	3 or 5-point Likert-type	Generic core instrument	Children self-report: 4–6 years (Kiddy) 7 to 13 years (Kid) 14–17 years (Kiddo) Patient proxy-report: 3 to 6 years (Kiddy) 7–17 years (Kid/Kiddo)
COHIP (Child Oral Health Impact Profile)	2007	To assess oral-facial wellbeing in school-age children	Children aged 8–15	English Spanish French Arabic Chinese Korean Dutch Amharic Persian	34 items 5 domains	5-point Likert-type	Oral health, functional wellbeing, social/emotional wellbeing, school environment, self-image	Patients and parents
COHIP-SF (Child Oral Health Impact Profile-Short Forms)	2012	To assess oral-facial wellbeing in school-age children with a short form	Children aged 8–15	English Japanese Indonesian Arabic Chinese German	19 items 5 domains	5-point Likert-type	Oral health, functional wellbeing, social/emotional wellbeing, school environment, self-image	Patients and parents
VPIQOL (Velopharyngeal Insufficiency Quality of Life)	2007	To assess alterations of QOL in children aged 5–17 years with VPI	5 to 17 years children with velopharyngeal insufficiency	English	48 items (43 for patients); 7 domains (6 for patients)	5-point Likert-type	Speech limitations, swallowing problems, situational difficulty, emotional impact, perception by others, activity limitations and caregiver impact	Patients and parents
VELO (VPI Effects on Life Outcomes instrument)	2012	To measure and follow QOL in patients with VPI	Velopharyngeal insufficiency	English Chinese Spanish Nepali Portuguese Dutch	23 items (26 for parents); 5 domains (6 for parents)	5-point Likert-type	Speech limitation, swallowing problems, situational difficulty, emotional impact, perception by others, caregiver impact	Patients and parents (Parent Proxy Assessment divided the patients with VPI into those 9 years or younger and those 10 years and older)
PVOS (Pediatric voice outcome survey)	2002	To measure the VR-QOL in the pediatric population	Children and adolescents with voice concerns specific to congenital- or neonatal-acquired lesions (sample age 2 to 18)	English Turkish	4 items	3 and 5-point Likert-type	NA	Parents
PVRQOL (Pediatric Voice-Related Quality-of-Life survey)	2006	To assess voice changes over time in the pediatric population	Pediatric with voice disorders (sample age 2 to 18)	English Arabic Turkish Brazilian Chinese Serbian	10 items	6-point Likert-type	NA	Parents
VHI (Voice Handicap Index)	1997	To quantify the psychosocial consequences of voice disorders	Adult voice disorder patients (sample mean age 52.3)	English Korean Czech Norwegian Croatian Japanese French Chinese Arabic Italian Portuguese Turkish Greek Spanish Hebrew Dutch German Swedish Russian Marathi Slovak Finnish Persian Serbian Danish Latvian Lebanese	30 items 3 domains	5-point Likert-type	Emotional, functional, physical	Patients
pVHI (Pediatric Voice Handicap Index)	2007	To quantify the impact of a voice disturbance on the child's social, emotional, and functional wellbeing	Dysphonia on the pediatric population (children younger than 3 were excluded)	English Persian Turkish Chinese Arabic Danish Italian Portuguese French Hebrew Korean Malayalam Spanish Dutch Polish	23 items 3 domains	5-point Likert-type	Emotional, functional, physical	Parents
VHI-9i (nine-item Voice Handicap Index)	2009	To quantify the psychosocial consequences of voice disorders with short form	Voice disorder patients	English Dutch French German Italian Portuguese Swedish	9 items	5-point Likert-type	Emotional, functional, physical	Patients
SWAL-QOL (Swallowing Quality of Life questionnaire)	2000	To measure treatment variations and treatment effectiveness.	Adult dysphagia patients (mean age was 65.8)	English French Norwegian German Persian Italian Dutch Swedish Chinese Greek	44 items 11 domains	5-point Likert-type	Burden, eating duration, eating desire, symptom frequency, food selection, communication, fear, mental health, social, fatigue, sleep	Patients; A close family member; Interviewers

*NA, not available, the instrument has no subdomains*.

Pediatric Voice Outcome Survey (PVOS) was the shortest instrument, with only 4 items. In contrast, KIDSCREEN-52 contains as many as 52 items and needs 15–20 min to complete. Most of the item numbers range from 10 to 40. Due to this, there were several types of instruments developed a short-form version to reduce the time burden (KIDSCREEN-27, KIDSCREEN-10, PedsQL 4.0, COHIP-SF, VELO, VHI-9i) (29, 34, 40–42).

In general, the majority of instruments were thoroughly discussed by experts and/or patients in the generating and modified procedure. The pilot study was common in a validation test. No noticeable gender difference was found. Except for 3 instruments (VELO, VHI, and SWAL-QOL), most instruments applied on patients with velopharyngeal inadequacy were designed only for children.

### Measures Assessment

#### Conceptual Model

Besides PVOS and PVRQOL instruments which intend to measure a single concept, most instruments clearly defined their construct and respective target population. The health definition from WHO and the conceptualization of health-related QOL were the most commonly used methods (21, 23, 24, 43).

#### Content Validity

Most of the earliest instruments in each type contained patient and expert participation. Only VHI was developed based on patients' opinions. The focus group was the most common method. During the modification of the original instruments, fewer studies involved target patients. Four instruments did not specify who developed the instrument (pVHI, PVOS, PVRQOL, and VHI-9i). Three instruments provided limited information about the development of items (KINDL, VPIQOL, and PVRQOL).

#### Reliability

Except for VPIQOL, all the other instruments were tested and demonstrated adequate reliability. VPIQOL did not provide any information about reliability determination (17).

#### Construct Validity

Construct validity dimension was one of the most demanding criteria to meet, especially for longitudinal validity. Only five instruments (KINDL, COHIP, VELO, PVOS, and PVRQOL) provided sufficient information to assess both test-retest reliability and responsiveness to change. Longitudinal validity was crucial for analyzing cohort study data and measuring intervention effect, which was particularly compromised by test-retest reliability and responsiveness validity. Test-retest reliability could guarantee the baseline stability, and responsiveness validity can measure the change before and after the intervention. The dimensionality was justified for 8 instruments (KIDSCREEN, PedsQL 4.0, KINDL, COHIP, COHIP-SF, VELO, VHI-9i, and SWAL-QOL) by factor analysis. In contrast, the criteria of convergent validity and known group validity were easy to meet. There were four instruments that failed to meet the convergent validity (VPIQOL, PVOS, VHI, and pVHI), and one instrument was unable to test the distinguish validity (PVRQOL).

#### Scoring and Interpretation

Compared with other dimensions, this dimension was the most difficult one to achieve. A total of 9 instruments clearly explained the scoring approach or algorithm (KIDSCREEN, PedsQL 4.0, KINDL, COHIP, VPIQOL, VELO, PVOS, PVRQOL, and VHI-9i), four of which described the plan for missing data (KIDSCREEN, PedsQL 4.0, KINDL, and COHIP). Five instruments provided information on how to interpret the scores (KIDSCREEN, PedsQL 4.0, KINDL, COHIP, and VHI-9i). The result also suggested that scoring and interpretation was the most neglected dimension during instrument development.

#### Respondent Burden and Presentation

In this dimension, all the instruments were available for public viewing. Seven instruments (KIDSCREEN, PedsQL 4.0, COHIP, COHIP-SF, VELO, VHI-9i, and SWAL-QOL) discussed the number of questions and retained a reasonable result. Five instruments (PedsQL 4.0, COHIP, COHIP-SF, VELO, and SWAL-QOL) described the literacy level.

**Figure 3:**
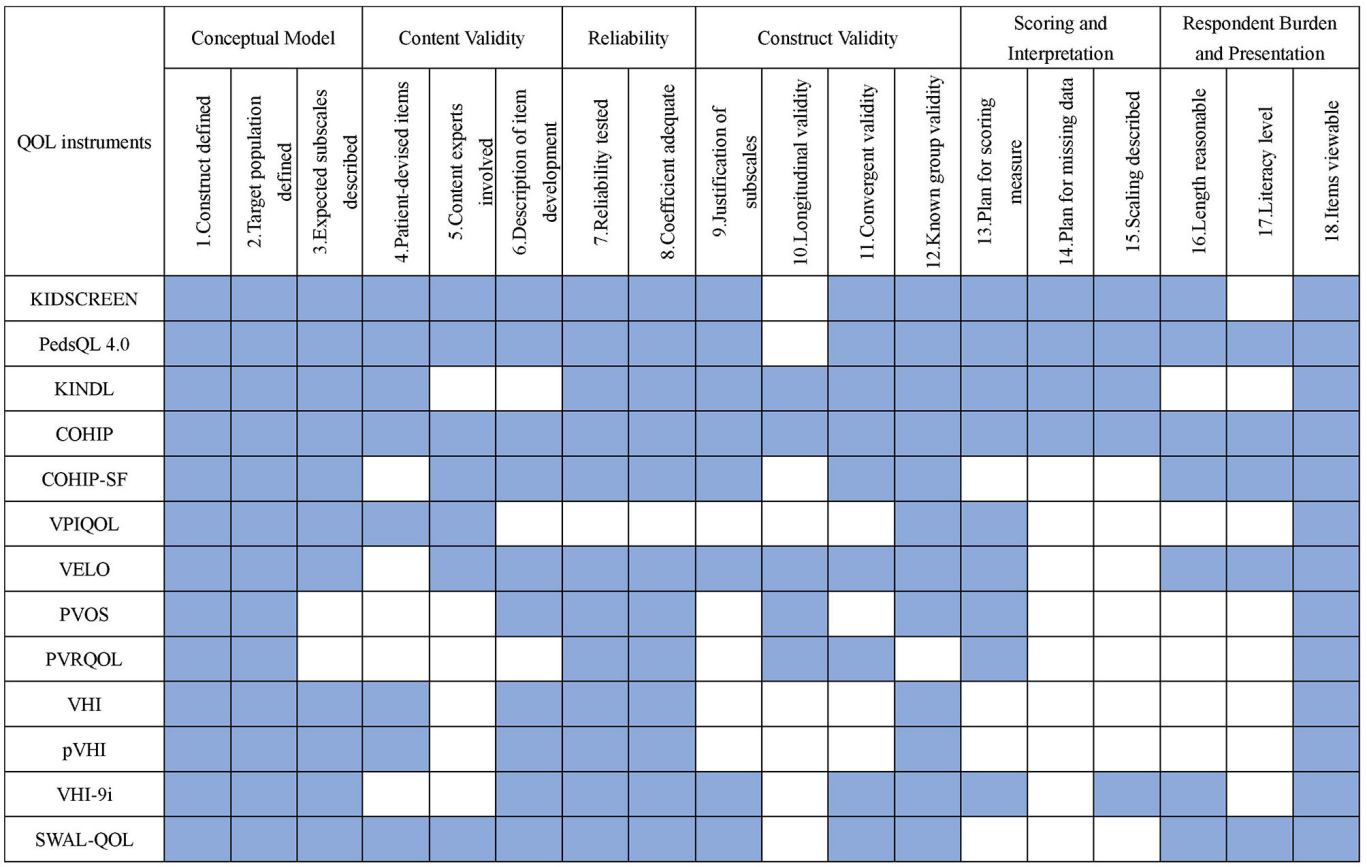
Summary comparison of measurement properties among identified instruments. Blue indicate the criterion is met. PedsQL 4.0, pediatric quality of life inventory 4.0 generic core scales; KINDL, german generic quality of life instrument for children; COHIP, child oral health ompact profile; COHIP-SF, child oral health ompact profile-short form; VPIEQOL, velopharyngeal insufficiency quality of life; VELO, velopharyngeal insufficiency effrets of the outcomes instrument; PVOS, pediatric voice outcome survey; PVRQOL, pediatric voice-related quality-of-life survey; VHI, voice handicap index; pVHI, pediatric voice handicap index; VHI-9i, 9-item voice handicap index; SWAL-QOL, swallowing quality of life questionnaire.

Among the 6 dimensions, reliability and conceptual model were the two easiest critera to meet, while construct validity and scoring and interpretation were two hardest to meet (Figure 3). COHIP (18/18) met the most criteria, followed by PedsQL 4.0 (17/18), KIDSCREEN (16/18), VELO (15/18), KINDL, and SWAL-QOL (14/18). VPIQOL (8/18) and PVRQOL (8/18) met the least criteria. Plan for missing data was the most challenging criteria for the instrument, and only four instruments mentioned it. Longitudinal validity, literacy level, and scaling description took the place of second hardest among all criteria, with only five instruments who fulfilled it. Except for VPIQOL, all the other instruments had been demonstrated with considerably good reliability.

## Discussion

The importance of QOL has been increasingly embodied (3). Similar to other chronic diseases, velopharyngeal inadequacy has a long course and is difficult to fully recover from. For a long time, surgical outcome evaluations have been patient-centered rather than patient-reported. QOL is a patient-reported outcome which can measure the experience of the target population and give patients the right to participate in the therapy. QOL assessment is also the only way to measure the patients' subjective feelings like depression, pain, satisfaction, and so on. Therefore, the treatment should pay attention to QOL improvement as much as surgical outcomes. Patients themselves, along with their caregivers, are enough to evaluate the QOL of the patients. Meanwhile, QOL should not be limited to the patient as circumstance of the disease influences the QOL of the whole family. In addition, there are some instruments pay attention to the QOL of family members (44, 45).

Results clearly showed the developmental characteristics, measurement aims, target population, item characteristics, and measurement properties of velopharyngeal inadequacy-related QOL instruments. Based on the results, this discussion section of the present systematic review tried to answer the following questions: (1) Who should be responsible for assessing the QOL of patients with velopharyngeal inadequacy? (2) How to choose the appropriate instrument? and (3) How can the QOL result apply for practice?

The opinion that the patient should evaluate his/her own QOL is a dominant one. Self-reported measurement can promote the treatment effects and encourage cooperation from the patients. It is also helpful and beneficial for clinical practice to more accurately reflect the patients' perception more accurately (46). One study showed that the parent-reported QOL outcomes could not provide further information regarding a child's QOL (47). Some others hold the opposite opinion in that the parent's view should be regarded as more important. Only parents can make a comprehensive and long-term evaluation of the consequence of illness (30).

The age of 6 marks the beginning of abstract thinking and self-concept (48). By the age of 11 or 12, children start to have a clear understanding of some complex emotions, such as worry, shame, and jealousy. Their self-concept acquires sophisticated dimensions, such as romantic appeal and popularity with peers. Children develop the concept of time at about the age of 8 when their recall period starts to lengthen and their understanding of the frequency of events begins to emerge. A. Jokovic et al. recommended age-specific QOL instruments for children aged 6–14. He proposed that instruments should be grouped into the following ages: 6–7-, 8–10-, and 11–14-year-olds (49). From the above, QOL is crucial and valuable no matter from the patient's self-report or the patient's parent-report. For school-aged children and adolescents, the self-report QOL instrument is the best choice. However, the caregivers are also the target population. Caregivers are usually dissatisfied with the children's QOL and therapy effect (31). It is also essential to “cure” the caregivers, make them have a reasonable expectation, and promote their cooperation. For children younger than 5 years old, the caregivers are highly recommended to be the ones to accomplish the assessment. Instruments, including PedsQL 4.0, KINDL, PVOS, PVRQOL, and pVHI, are great options. For children aged 5–7 years old, choice can be made from KIDSCREEN, PedsQL 4.0, andKINDL for self-report. For school-age children (8 and above), VPIQOL, VELO, and COHIP-SF can be used for self-report.

Besides the age of the target population, the research objective should be one important factor to consider for choosing instruments. If we want to compare general and oral health between patients with velopharyngeal inadequacy and healthy people, a generic instrument like KIDSCREEN, PedsQL 4.0, KINDL, COHIP, or COHIP-SF could be better. VPIQOL and VELO are designed for measuring the specific VPI-related health problems. If we focus on the voice problem, we can choose VHI, pVHI, PVOS, or PVRQOL. If we focus on the swallowing problem, SWAK-QOL is the right choice. If we want to measure the therapeutic change, we have to choose an instrument that has a good test-retest reliability and responsiveness validity, such as KINDL, COHIP, VELO, PVOS, and PVRQOL.

Language is another influencing factor for choosing an instrument. Most of the instruments are developed in English. If we want to translate the English version instrument and use it, we would first have to do the validation research in the target population. Applying the transferred and validated instruments could save time and labor. The earlier developed instruments have a bigger chance of being translated and tested with various language versions. Among these instruments, PedsQL 4.0 has the largest number of language versions which could be widely applied in most countries (Table 3). In addition, if we want to apply an instrument to a rural area, the literacy level should be taken into account.

Time burden can limit the clinical application. Therefore, time burden plays a crucial role in choosing instruments. With respect to comprehensiveness, generally speaking, long instruments with more items could provide more information. In terms of acceptance and practicability, short instruments are more appropriate to use. PVOS is the shortest instrument, with only 4 items, compared to the longest instrument, KIDSCREEN, with 52 items. Pilot tests can be applied before deciding on the instrument, like recording the time and assessing the feedback from the target population. The sampling set and the number of working staff should also be considered. It is difficult for a busy clinic or other hand-shorted places to handle a long instrument.

The first included article was published in 2004 with the PVOS instrument, which aimed to assess the outcome of surgery for velopharyngeal insufficiency (50). In contrast, few studies have recently applied PVOS. This might be due to its simplicity. The VELO instrument has the dominant place in the recent 3 years (20, 51–61), followed by the type 8 family (VHI, pVHI, and VHI-9i) (57, 61–64). The VELO instrument is specially designed for patients with velopharyngeal insufficiency who accounted for the majority of patients with velopharyngeal inadequacy, thereby enabling its widespread. The VHI instrument has the longest history, which indicates the primary place of voice-related QOL for velopharyngeal inadequacy QOL.

Finally, how can the QOL result be applied to practice? QOL result is one of the therapeutic effect indexes which can indicate the health outcome. The outcome is likely to be influenced by patient and medical treatment factors. After adjusting the patient factors as confounders, the variation of outcomes can be attributed to the difference of treatment effect, which is important for treatment assessment, comparison, and improvement (3, 65–67). Apart from this, the distribution of the health outcomes of patients with velopharyngeal inadequacy can be used for estimating health service demands to provide evidence for health resource allocation.

Some questions, such as the following, still remain unexplored and can be used as directions for future studies of VPI-related QOL measures: (1) What's the relationship between QOL results and other therapeutic indexes? (2) How big is the difference between patients with velopharyngeal inadequacy and their caregivers? Does the difference change with age? and (3) How many changes in the scores can suggest the treatment is effective?

## Conclusion

Quality of Life (QOL) is an essential index to measure treatment effects. Patient self-reported assessment and caregiver proxy assessment are both valuable. The choice of QOL measure instrument should be made according to research aim, target population, language requirement, time, and labor resources.

## Data Availability Statement

The original contributions presented in the study are included in the article/Supplementary Material, further inquiries can be directed to the corresponding author/s.

## Author Contributions

NC and HH contributed to the collection of data, writing, and revising the article. NC analyzed the data. BS and HH supervised the research. All authors contributed to the article and approved the submitted version.

## Funding

This work was supported by the Research and Develop Program, West China Hospital of Stomatology Sichuan University grant to HH (RD-02-202107) and the National Natural Science Foundation of China grant to BS (81974147).

## Conflict of Interest

The authors declare that the research was conducted in the absence of any commercial or financial relationships that could be construed as a potential conflict of interest.

## Publisher's Note

All claims expressed in this article are solely those of the authors and do not necessarily represent those of their affiliated organizations, or those of the publisher, the editors and the reviewers. Any product that may be evaluated in this article, or claim that may be made by its manufacturer, is not guaranteed or endorsed by the publisher.
