# Erratum: Velopharyngeal Inadequacy-Related Quality of Life Assessment: The Instrument Development and Application Review

**DOI:** 10.3389/fsurg.2022.903725

**Published:** 2022-04-12

**Authors:** Frontiers Production Office

**Affiliations:** Frontiers Media SA, Lausanne, Switzerland

**Keywords:** velopharyngeal inadequacy, quality of life, instruments, patient-report outcomes, patient-report outcome questionnaire

Due to a production error, the article was mistakenly published as the article type “Original Research” instead of “Systematic Review”.

The publisher apologizes for this mistake. The original article has been updated.

## Publisher's Note

All claims expressed in this article are solely those of the authors and do not necessarily represent those of their affiliated organizations, or those of the publisher, the editors and the reviewers. Any product that may be evaluated in this article, or claim that may be made by its manufacturer, is not guaranteed or endorsed by the publisher.

