# Supplementary material for: Velopharyngeal Inadequacy-Related Quality of Life Assessment: The Instrument Development and Application Review
Source: Front Surg. 2022 Mar 8;9:796941. doi: 10.3389/fsurg.2022.796941 (PMC8988257; doi:10.3389/fsurg.2022.796941)
Supplement: Supplementary file 1 [file Table_1.DOCX]

##### Supplemental Materials

**Supplemental Table 1. Population characteristics and setting, involved in the development of QOL instruments.**

| Article | Instrument | Study Population | Setting | Number of participants | Distribution of pathology | Mean age (SD), [Range] | Female (%) | Country |
| --- | --- | --- | --- | --- | --- | --- | --- | --- |
| Ulrike Ravens-Sieberer^1^ | KIDSCREEN | Families who had agreed in 12 European countries | A European project “Screening and Promotion  for Health-related Quality of Life in Children and Adolescents - A European Public Health Perspective” including 13 European countries | 22295 | NA | Children 9.57 [8-11]  Adolescents 14.27 [12-18] | Children: 51.3  Adolescents: 53.1 | European Commission |
| JAMES W. VARNI^2^ | PedsQL 4.0 | English- or Spanish-speaking 2-18 parent and healthy children and pediatric patients with acute or chronic health conditions | State’s Children’s Health Insurance Program (SCHIP), throughout the State of California | children ages 5 to 18 years (n=963) and parents of children ages 2 to 18 years (n= 1,629), with 1,677 participants accrued overall | Chronically ill 683, acutely ill 207, healthy children 730 | 9.3 (4.37) [2.0-18.8] | 49.5 | USA |
| Ulrike Ravens-Sieberer & Monika Bullinger^3^ | KINDL | Aged 10–16 years with a diagnosis of either juvenile diabetes or bronchial asthma | A German children’s Hospital and the German National Health Interview and Examination Survey for Children and Adolescents (KiGGS) | 145 | Juvenile diabetes or bronchial asthma | 13.2 (2.7) [10-16] | 51 | German |
| Broder HL^4^ | COHIP | Patients seeking pediatric dental, orthodontic treatment, or craniofacial care and being between the ages of 8 and 15 | Pediatric and Orthodontic clinics at the University of Medicine and Dentistry in Newark, NJ, the Pediatric and Orthodontic clinics at New York University School of Dentistry, the Center for Reconstructive Surgery at New York University Medical Center in NYC, and McGill University Hospital and the pediatric and orthodontic clinics at McGill University, Montreal, Canada. | Face validity 1: 144 caregivers;  Item impact 1: 155 children and their caregivers;  Face validity 2: 50 children and 55 caregivers  Item impact 2: 178 children and their caregivers;  Factor analyses: 419 children | Seeking pediatric dental,  orthodontic treatment, or craniofacial care | Item impact 1: children 11.8 (1.5)  Item impact 2: 12.1 (1.4)  Factor analyses: 11.8 (1.5) | Item impact 1: 55  Item impact 2: 51.7  Factor analyses: 50.5 | USA, Canada |
| Hillary L. Broder^5^ | COHIP-SF | Participants with  craniofacial anomalies (CFAs) between 7-18 years of age | Population-based health study of agricultural workers in Mendota, California; and postgraduate clinic at New York University (NYU) College of Dentistry. | Pediatric: 205  Orthodontic: 108  Craniofacial: 863 | Pediatric:205 Orthodontic: 108 Craniofacial: 863 | Pediatric: 11.8 (2.9) [7-17]  Orthodontic: 12.7 (2.0) [9-17]  Craniofacial: 11.9 (3.0) [7-19] | Pediatric: 45  Orthodontic: 50  Craniofacial: 44 | USA |
| Lucy Barr^6^ | VPIQOL | VPI patients with 5 to 17 years old and their parents | Primary Children’s Medical Center (PCMC) in Salt Lake City, Utah and Columbia University, New York | 29 | Velopharyngeal insufficiency | 8.7 | 48.3 | USA |
| Jonathan R. Skirko, 2012^7^ | VELO | Patients with VPI and their parents | Department of Otolaryngology–Head and Neck Surgery, Seattle Children’s Hospital, Washington | 29 | NA | 8.7 (3.0) [5-17] | 48.3 | USA |
| Christopher J. Hartnick^8^ | PVOS | caregivers of children or adolescents requiring tracheotomies or who had required tracheotomies in the past | Cincinnati Children’s Hospital Medical Center, Cincinnati, Ohio | 108 | Parents and caregivers of children and adolescents who  have undergone tracheotomy | NA | NA | USA |
| Mark E. Boseley^9^ | PVRQOL | Children aged 2 through  18 years having a variety of otolaryngological problems | Outpatient office practice of Departments of Otolaryngology, Massachusetts Eye and Ear Infirmary and Children’s Hospital Boston, Boston, Mass | 120 | VPI 4  Dysphonia 10  Adenotonsillar hypertrophy 29  Otitis Media 20  Sinus Disorder 9  Other 39 | [2-18] | 40 | USA |
| Barbara H. Jacobson, 1997^10^ | VHI | Patients with voice disorders | Voice Clinic, Henry Ford Hospital and Health Sciences Center, Detroit, Michigan | 63 | 21 mass lesions, 17 neurogenic, 17 laryngectomy, 5 musculoskeletal tension, 3 inflammatory, 2 atypical | 49 (18) [NA] | 60.32 | USA |
| Karen B. Zur^11^ | pVHI | Guardians of children presenting for a voice  evaluation pre- or post-laryngotracheal reconstruction | Department of Otolaryngology, Cincinnati Children’s Hospital Medical Center, Cincinnati | 33 | subglottic stenosis secondary to  prolonged intubation | 11 [4-21] | 48.89 | USA |
| T. Nawka^12 13^ | VHI-9i | Patients sought therapeutic advice because of voice complaints | From 8 countries | 1052 | Vocal dysfunction without organic vocal fold changes; Vocal fold nodules; Structural lesions of the epithelium and lamina propria; Unilateral paresis of the vocal fold; Laryngitis | 45 [12-86] | 66 | Belgium, France, Sweden, Germany, Italy, The Netherlands, Portugal, and the USA |
| Colleen A. McHorney ^14 15^ | SWAL-QOL | Patients with mechanical or neurologic oropharyngeal dysphagia | Center for Health Services Research and Management, University of Kentucky Medical Center, and the HSR&D Program at the Lexington VA Medical Centers, Lexington, Kentucky | 1.Item development: 52 patients and 15 caregivers  2.Item reduction: 106 patients | 109 Cancer, 61 Vascular diseases, 49 Degenerative neurologic diseases, 36 other neurologic diseases, 23 obstructive respiratory diseases, 15 trauma, 17 chronic medical conditions, 4 dementia, 26 other reasons, 46 unknown reason | 66.1 (13.17) [NA] | 21.5 | USA |

1. Ravens-Sieberer U, Gosch A, Abel T, et al. Quality of life in children and adolescents: a European public health perspective. *Soz Praventivmed* 2001;46(5):294-302. doi: 10.1007/bf01321080 [published Online First: 2002/01/05]

2. Varni JW, Seid M, Kurtin PS. PedsQL 4.0: reliability and validity of the Pediatric Quality of Life Inventory version 4.0 generic core scales in healthy and patient populations. *Med Care* 2001;39(8):800-12. doi: 10.1097/00005650-200108000-00006 [published Online First: 2001/07/27]

3. Ravens-Sieberer U, Bullinger M. Assessing health-related quality of life in chronically ill children with the German KINDL: first psychometric and content analytical results. *Qual Life Res* 1998;7(5):399-407. doi: 10.1023/a:1008853819715 [published Online First: 1998/08/06]

4. Broder HL, McGrath C, Cisneros GJ. Questionnaire development: face validity and item impact testing of the Child Oral Health Impact Profile. *Community Dent Oral Epidemiol* 2007;35 Suppl 1:8-19. doi: 10.1111/j.1600-0528.2007.00401.x [published Online First: 2007/08/31]

5. Broder HL, Wilson-Genderson M, Sischo L. Reliability and validity testing for the Child Oral Health Impact Profile-Reduced (COHIP-SF 19). *J Public Health Dent* 2012;72(4):302-12. doi: 10.1111/j.1752-7325.2012.00338.x [published Online First: 2012/04/28]

6. Barr L, Thibeault SL, Muntz H, et al. Quality of life in children with velopharyngeal insufficiency. *Arch Otolaryngol Head Neck Surg* 2007;133(3):224-9. doi: 10.1001/archotol.133.3.224 [published Online First: 2007/03/21]

7. Skirko JR, Weaver EM, Perkins J, et al. Modification and evaluation of a Velopharyngeal Insufficiency Quality-of-Life instrument. *Arch Otolaryngol Head Neck Surg* 2012;138(10):929-35. doi: 10.1001/2013.jamaoto.122 [published Online First: 2012/10/17]

8. Hartnick CJ. Validation of a pediatric voice quality-of-life instrument: the pediatric voice outcome survey. *Arch Otolaryngol Head Neck Surg* 2002;128(8):919-22. doi: 10.1001/archotol.128.8.919 [published Online First: 2002/08/07]

9. Boseley ME, Cunningham MJ, Volk MS, et al. Validation of the Pediatric Voice-Related Quality-of-Life survey. *Arch Otolaryngol Head Neck Surg* 2006;132(7):717-20. doi: 10.1001/archotol.132.7.717 [published Online First: 2006/07/19]

10. Jacobson BH, Johnson A, Grywalski C, et al. The Voice Handicap Index (VHI). *American Journal of Speech-Language Pathology* 1997;6(3):66-70. doi: 10.1044/1058-0360.0603.66

11. Zur KB, Cotton S, Kelchner L, et al. Pediatric Voice Handicap Index (pVHI): a new tool for evaluating pediatric dysphonia. *Int J Pediatr Otorhinolaryngol* 2007;71(1):77-82. doi: 10.1016/j.ijporl.2006.09.004 [published Online First: 2006/10/19]

12. Caffier F, Nawka T, Neumann K, et al. Validation and Classification of the 9-Item Voice Handicap Index (VHI-9i). *J Clin Med* 2021;10(15) doi: 10.3390/jcm10153325 [published Online First: 2021/08/08]

13. Nawka T, Verdonck-de Leeuw IM, De Bodt M, et al. Item reduction of the voice handicap index based on the original version and on European translations. *Folia Phoniatr Logop* 2009;61(1):37-48. doi: 10.1159/000200767 [published Online First: 2009/02/11]

14. McHorney CA, Bricker DE, Robbins J, et al. The SWAL-QOL outcomes tool for oropharyngeal dysphagia in adults: II. Item reduction and preliminary scaling. *Dysphagia* 2000;15(3):122-33. doi: 10.1007/s004550010013 [published Online First: 2000/06/06]

15. McHorney CA, Bricker DE, Kramer AE, et al. The SWAL-QOL outcomes tool for oropharyngeal dysphagia in adults: I. Conceptual foundation and item development. *Dysphagia* 2000;15(3):115-21. doi: 10.1007/s004550010012 [published Online First: 2000/06/06]
